# Physical durability and insecticidal activity of long-lasting insecticidal nets in Cruzeiro do Sul, Brazil

**DOI:** 10.1038/s41598-024-59172-7

**Published:** 2024-04-19

**Authors:** Ana Cecília Feio-dos-Santos, Crissiane C. Reis, Izis M. C. Sucupira, Audrey Lenhart, Márcia M. M. Santos, Ediane R. Reis, Ediclei Lima do Carmo, Simone Daniel, Oscar M. Mesones Lapouble, Alexandre Macedo de Oliveira, Marinete M. Povoa

**Affiliations:** 1 Pós-Graduação em Biologia de Agentes Infecciosos e Parasitários, Universidade Federal do Pará, Belém, Pará 66075-110 Brazil; 2 Laboratório de Entomologia de Malária, Seção de Parasitologia, Instituto Evandro Chagas/SVSA/MS, Ananindeua, Pará CEP 67030-000 Brazil; 3 Division of Parasitic Diseases and Malaria, Entomology Branch, Centers for Disease Control and Prevention (CDC), 1600 Clifton Road, Atlanta, GA 30329 USA; 4 Seção de Parasitologia, Instituto Evandro Chagas/SVSA/MS, Ananindeua, Pará CEP 67030-000 Brazil; 5 Hospital Juruá, Av. 25 de Agosto, 2151, Cruzeiro do Sul, Acre Brazil; 6 Pan American Health Organization/World Health Organization Office in Suriname, Henck Arronstraat #60, Paramaribo, Suriname; 7 Division of Parasitic Diseases and Malaria, Malaria Branch, Centers for Disease Control and Prevention, Atlanta, GA USA

**Keywords:** Malaria, Vector control, Long-lasting insecticidal nets, Entomology, Malaria

## Abstract

Vector control is one of the principal strategies used for reducing malaria transmission. Long-lasting insecticidal bed nets (LLINs) are a key tool used to protect populations at risk of malaria, since they provide both physical and chemical barriers to prevent human-vector contact. This study aimed to assess the physical durability and insecticidal efficacy of LLINs distributed in Cruzeiro do Sul (CZS), Brazil, after 4 years of use. A total of 3000 LLINs (PermaNet 2.0) were distributed in high malaria risk areas of CZS in 2007. After 4 years of use, 27 ‘rectangular’ LLINs and 28 ‘conical’ LLINs were randomly selected for analysis. The evaluation of physical integrity was based on counting the number of holes and measuring their size and location on the nets. Insecticidal efficacy was evaluated by cone bioassays, and the amount of residual insecticide remaining on the surface of the LLINs was estimated using a colorimetric method. After 4 years of use, physical damage was highly prevalent on the rectangular LLINs, with a total of 473 holes detected across the 27 nets. The upper portion of the side panels sustained the greatest damage in rectangular LLINs. The overall mosquito mortality by cone bioassay was < 80% in 25/27 rectangular LLINs, with panel A (at the end of the rectangular bednet) presenting the highest mortality (54%). The overall mean insecticide concentration was 0.5 µg/sample, with the bednet roof containing the highest average concentration (0.61 µg/sample). On the conical LLINs, 547 holes were detected, with the bottom areas sustaining the greatest damage. The cone bioassay mortality was < 80% in 26/28 of the conical LLINs. The mean insecticide concentration was 0.3 µg/sample. After 4 years of use, the insecticidal efficacy of the LLINs was diminished to below acceptable thresholds.

## Introduction

The Brazilian Amazon continues to be the main malaria endemic area in Brazil, representing a challenge for public health authorities^1^. Since the beginning of the 2000’s, the Brazilian Ministry of Health (MOH) adopted prompt diagnosis and treatment as the principal strategies for reducing mortality and case severity^2^. Measures for vector control including the use of long-lasting insecticidal bed nets (LLINs) were implemented in highly endemic areas. The LLINs are distributed by the Brazilian MOH in two styles, conical for single or double beds and rectangular for hammocks^3,4^.

LLINs are an important malaria control tool for diminishing human/vector contact and are commonly provided to populations living in areas of malaria transmission risk^3,5^. Three municipalities in the Brazilian Amazon Region were the first to use LLINs as a strategy to control malaria: Cruzeiro do Sul, Mancio Lima and Rodrigues Alves. In 2007, both LLIN styles (conical and rectangular) were distributed in Cruzeiro do Sul (CZS) after a major malaria outbreak in 2006 with an API (Annual Parasite Index) of 571.5 malaria cases per 1000 inhabitants^6,7^. The main malaria vector in CZS, *Anopheles darlingi*, is present in both the intra- and peridomicile and its haematophagic activity occurs mainly between 6 and 9pm^8^.

In African countries, LLINs and indoor residual spraying (IRS) are the main malaria vector control measures used^9^. The information arising from experiences in Africa, including studies related to LLIN durability and effectiveness, has informed LLIN procurement decisions elsewhere, including determining the frequency with which they should be replaced^10,11^. In Brazil, data about malaria vectors, transmission dynamics and evaluations of vector control tools are comparatively scarce, and there are no previous studies evaluating long-term LLIN durability^12^. Thus, this study aimed to evaluate, for the first time, the physical durability and insecticidal efficacy of conical and rectangular LLINs after 4 years of use across CZS municipality.

## Results

### LLIN physical integrity

Only 1/27 of all rectangular LLINs analyzed had no detectable physical damage after 4 years of use. A total of 473 holes were found on the rectangular LLINs, resulting in a median of 8 holes (ranging from 0 to 63 holes; mean of 17.5 holes), with 51.8% in good condition, with pHI < 24. Damage was evenly distributed amongst the panels, with no statistically significant differences detected. Over half of the LLINs (16/27) had rips along the seams between the panels, resulting in a total of 40 rips, 70% of which were size 2. The area of greatest damage to the rectangular LLINs was along the top of the side panels of the nets, in about 45% of all rectangular nets.

Only 2/28 of the conical LLINs analyzed showed no evidence of physical damage after 4 years of use. A total of 547 holes were found on the conical LLINs, with a median of 13.5 holes/bednet (ranging from 0 to 70 holes; mean of 19.5), with only 28.6% in a good condition, with pHI < 24. The area of greatest damage was the bottom of the nets, in about 78% of all conical nets. Damage was evenly distributed amongst the panels, and on two of the conical nets, three rips were found along the seams between panels.

**Figure 1:**
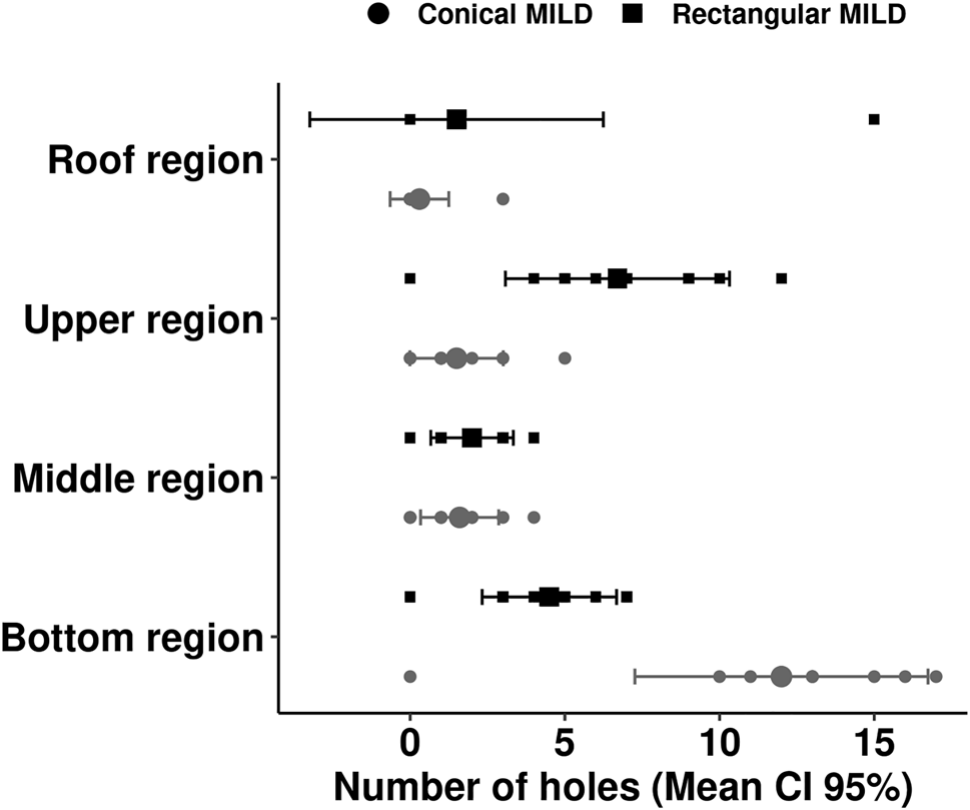
Mean number of holes by location on rectangular and conical LLINs The largest shapes correspond to means and bars 95% CI).

No significant differences were detected related to the number of holes between the LLIN types (*p* = 0.684). However, rectangular nets sustained more damage at the top and conical nets at the bottom (Fig. 1), showing significant statistical differences (Table 1).

**Table 1:** Comparison among the number of physical damaged regions between conical and rectangular LLINs.

Sidak's multiple comparisons test Conical × rectangular LLINs	Mean Diff	Confidence interval	Adjusted *p* value
Roof region (R)	− 1.2	− 3.829 to 1.429	0.6652
Upper region (U)	− 5.2	− 7.829 to − 2.571	< 0.0001*
Middle region (M)	− 0.4	− 3.029 to 2.229	0.9910
Bottom region (B)	7.5	4.871 to 10.13	< 0.0001*

### WHO cone bioassays

**Figure 2:**
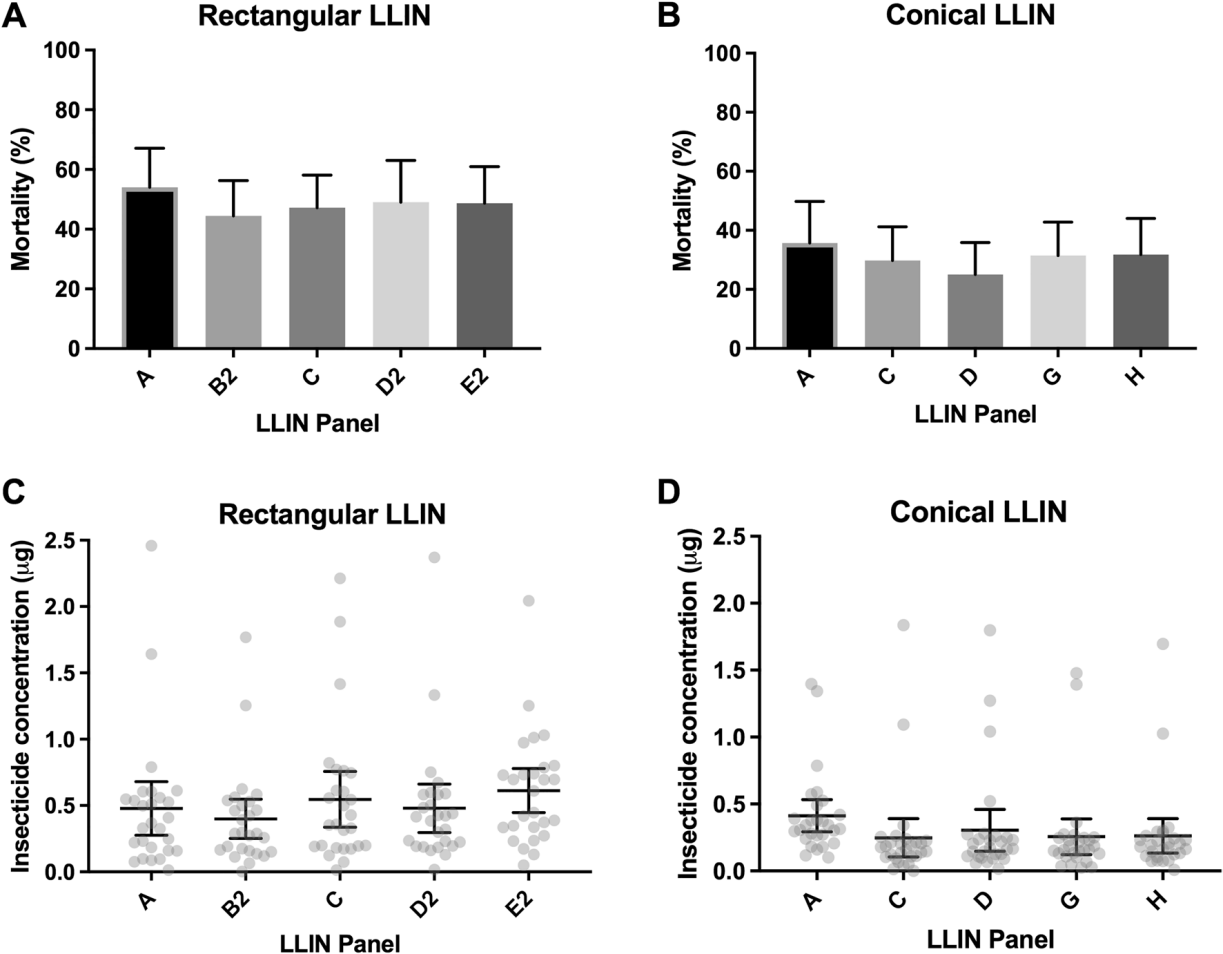
Mean bioassay mortality and deltamethrin surface insecticide concentration by panel of rectangular (**A**,**C**) and conical (**B**,**D**) LLINs.

In 25/27 of the rectangular nets tested, the mortality in cone bioassays was < 80%. The median mortality of the 27 LLINs was 60% (ranging from 4 to 92% - Supplementary Table 1). When mortality was analyzed using the same 5 panels tested by both WCT and CFT (A, B2, C, D2 and E2), we did not find statistically significant differences between the panels in terms of bioassay mortality (*p* = 0.706) (Fig. 2). A hundred per cent of the control mosquitoes survived in in all bioassays.

In 26/28 conical LLINs tested, the mortality was < 80%. The median mortality was 25.5% (ranging from 0 to 96% - Supplementary Table 2). When mortality was analyzed using the same 5 panels tested by both WCT and CFT (A, C, D, G and H), no statistically significant differences were detected among panels in terms of bioassay mortality (*p* = 0.334) (Fig. 2).

### Colorimetric test (CFT)

The median concentration of surface insecticide on rectangular LLINs was 0.39 μg/sample (range: 0.003–2.458 µg/sample; mean of 0.5 µg/sample - Supplementary Table 1). Of the nets tested, 40.7% (n = 11) presented a mean concentration < 0.35 μg/sample, which was a threshold value previously proposed, below which the net could be considered as failed^13^. In the analysis of the sides, the roof (piece E2) showed a significantly higher concentration of insecticide, with 0.61 μg/sample (95% CI 0.446–0.779; *p* < 0.0001) followed by side C, with 0.54 μg/sample (95% CI 0.336–0.756) (Fig. 2).

The median concentration of surface insecticide on conical LLINs was 0.20 µg/sample (range: 0.00016 to 1.84 µg/sample, mean of 0.33 µg/sample - Supplementary Table 1). Of the nets tested, 85.7% (n = 24) had a mean concentration < 0.35 µg/sample. In the analysis of the panels, panel “A” contained significantly higher surface insecticide (0.41 µg/sample; 95% CI 0.29–0.53), in comparison to the other panels (*p* < 0.0001) (Fig. 2).

### Comparison between WCT and CFT

**Figure 3:**
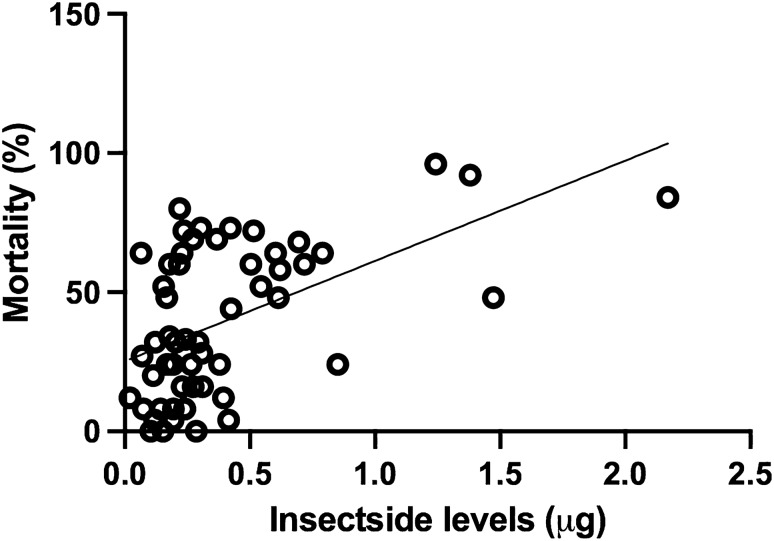
Results of linear regression of data from colorimetric tests (µg/sample of insecticide) versus mortality in cone bioassays. Best fitting regression line shows a positive and significant relationship (r^2^ = 0.2705, *p* < 0.0001).

In order to test the relationship between cone bioassay mortality and surface insecticide concentration from the CFT, we performed a logistic regression that showed a positive and statistically significant relationship (r^2^ = 0.2705; *p* < 0.0001), regardless of the type of mosquito net (rectangular or conical) (Fig. 3).

## Discussion

The results of this study showed that after 4 years of use, nearly all (96.4%) of the LLINs (PermaNet 2.0—100 denier polyester-based) had sustained some degree of physical damage, but 40% of the all nets were still effective as a physical barrier (pHI < 24) The LLIN damages reported here are similar to those reported elsewhere. LLINs evaluations using PermaNet 2.0 after 3 years, showed in Ghana 52.1%^10^ and in Senagal 50% of the physical damages were size 1 holes (0.5–1.0 cm in diameter)^14^. In Laos, it was reported that 40% of LLINs presented physical damage after 2 years of use 150 denier polyethylene-based^15^ as well in Zanzibar, using the same brand and type of fabric of LLINs, high damage was observed in 68% of LLINs after 3 years of use^16^. In Cruzeiro do Sul, the LLINs Permanet 2.0 were in a good condition after 4 years of use, although Killian et al.^17^ had showed a slighter advantage of polyethylene-based LLINs on polyester-based, ones. In the same way, Allan et al.^18^ demonstrated that polyester-based had 4.22 times more chances of having a poor or very poor condition than polyethylene-based LLINs, and Skovmand and Bosselmann^19^ showed that multifibre polyester nets are weaker than commercialized mono-fibre polyethylene nets.

On the rectangular nets, the greatest damage was found in the upper region of the side panels. In the upper region, the damage may be explained by the local practice of placing a "rod" (wooden stick) along the upper portion of the side panels so that the nets are well-stretched, which can cause tears at the top. At the bottom, damage was likely due to increased manipulation due to being the place of entry and exit of people from the mosquito nets. On the conical LLINs, the greatest damage was recorded on the lower region, and this is likely because it is the site of contact with the bed platform (typically made of unfinished wood). In addition, people have the habit of knotting the nets to store them raised during the day, or fix them to walls with sharp materials, which can also damage them. Our findings highlight the importance of reminding the population about correct LLIN use to avoid compromising their durability (Supplementary file 1).

The cone bioassay tests found that the nets fell below the acceptable measure of bioefficacy after 4 years of use, since mortality was lower than 80% for nearly all the nets tested. However, it was not possible to determine when the LLINs began to lose their bioefficacy since only a single evaluation was performed. It is worth to note that LLINs were not designed to last 4 years but only 3. Ideally, annual evaluations to monitor the loss of insecticidal efficacy would more accurately identify the duration of the insecticidal effect of the LLINs and could be used to guide replacement strategies with greater precision. As previously observed in Laos, LLINs may need to be replaced more frequently than every 3 years^15^, and research from Ethiopia documented that the insecticidal efficacy of LLINs decreased over time^20^. In a study from Nicaragua on the durability of rectangular LLINs treated with deltamethrin, the median cone bioassay mortality after 36 months of use was only 2%^21^. Our results showed a median mortality of 33.4% (range: 0 to 96%) for both types of LLINs analyzed, after 4 years of use. When analyzing only rectangular nets, the median mortality was 60% (range: 4 to 92%). Despite the notable differences in bioefficacy compared to the study carried out in Nicaragua, our data were in alignment with the results of the correlation between colorimetric assays and bioassays conducted in Nicaragua, which also showed a positive correlation between the surface deltamethrin levels and mosquito mortality (Fig. 3).

Several factors may reduce the insecticidal efficacy of LLINs, such as frequent and inadequate washing, or drying in direct sunlight^15^, although, in this study area, most net users had informally reported following all washing and drying guidelines.

The colorimetric test detected significant differences between the surface insecticide concentrations found on the rectangular LLINs (0.5 µg/sample) compared to the conical LLINs (0.3 µg/sample). In addition, a statistically significant difference was observed between mortality in the cone bioassays between rectangular (49%) and conical (31%) LLINs. Green et al.^13^ demonstrated that LLINs used in Africa containing less than 0.35 µg/LLINs sample—10 mg/m2 that was 15% of the original insecticide concentration indicated that the LLIN had failed. However, in this study, of the 17 rectangular nets for which the value of the CFT was above 0.35 µg/sample, 14 presented mortality lower than 80% in the cone bioassay, and of the 4 conical nets with CFT above 0.35 µg/sample, 2 did not achieve 80% mortality in the biaossays. We cannot be sure if the apparent lack of sensitivity of the cutoff value of 0.35 µg/sample could be attributed to biological differences between the African and Brazilian anophelines, thus, further research is needed to evaluate the CFT cutoff value for *Anopheles* species in Brazil. However, the results of the cone bioassay and the colorimetric test were consistently well-associated, since when compared across the two types of nets used, no significant differences were detected. The colorimetric test was simple and fast, and has the additional benefit of not requiring live mosquitoes, which makes it a highly desirable method for this type of evaluation in Brazil where colonizing *An. darlingi* is particularly challenging. Furthermore, it is important to mention that the CFT measures the available insecticide on the surface, which is expected to be a more significant measure of bioefficacy than total insecticide content, as is often used for durability monitoring.

In the analysis of the panels of the rectangular LLINs, the position with the highest insecticide concentration was the roof, and on the conical LLINs, side A (upper side). Both are positions of little contact with people or objects. While one or two sides may retain disproportionately higher insecticide concentrations, this does not necessarily mean that the mosquito net as a whole provides an effective chemical barrier. It has been observed in studies in Africa that the levels of deltamethrin on the surface of an LLIN may diminish disproportionately over time, with a half-life of deltamethrin on the bottom portion of 2.4 months and on the upper portion of 5.6 months^13^. This suggests that the retention of the insecticide is closely related to the handling of the LLINs. As for the bottom side, independent of the user population, it likely presents a lower concentration of insecticide over time because it is the area of greatest manipulation (people entering/exiting, contact with bedding and other objects)^13^. However, Parker and cols using laboratory^22^ and field tests^23^ showed that it is possible for LLINs to still be effective if one of the sides is still impregnated with insecticides, especially on the roof, where these authors show a greater number of mosquitoes close to the nets. Our results showed that the roof of rectangular mosquito nets (Side E) and the top of conical mosquito nets (Side A) had a higher concentration of deltamethrin, which can determine the effectiveness of these mosquito nets even after 4 years of use.

In the municipality of Cruzeiro do Sul, the State Government implemented a set of actions to control the transmission of malaria, which involved investments in early diagnosis and treatment, recruitment of field personnel for entomological control, and implementing the use of LLINs and environmental control of breeding sites^6^. Those measures were implemented during a malaria outbreak, which resulted in a 67% reduction in the number of cases^4^. In the state of Amazonas, Brazil, it was observed that the effectiveness of LLINs was related to the socio-cultural profile of the population and the capacity of the local health teams to sensitize the community to appropriate LLIN use^24^. In contrast, in Rondônia State, no statistically significant differences in API were detected 1 year after LLIN installation when compared to municipalities without LLINs. However, the installation of LLINs in Rondônia was not accompanied by actions to evaluate their use, effectiveness, or durability, highlighting the need for further evaluations of this nature, with the aim of generating a body of evidence to support their widespread distribution in the Amazon Region^25,26^.

It should be noted that in CZS the number of malaria cases has been increasing since 2012, and that the increase in cases caused by P. falciparum is particularly worrying (35.4% in 2020)^4^. As a result, the health authorities continue to rely on LLINs as a key tool to prevent malaria. In addition, the potential development of deltamethrin resistance in the local mosquitoes is an important point to be considered and will need to be further evaluated and taken into account when selecting ongoing vector control tools. The data reported here fill an important gap regarding the effectiveness of a key malaria vector control tool in Brazil^12^, which can provide guidance for future malaria control program actions.

## Methods

**Figure 4:**
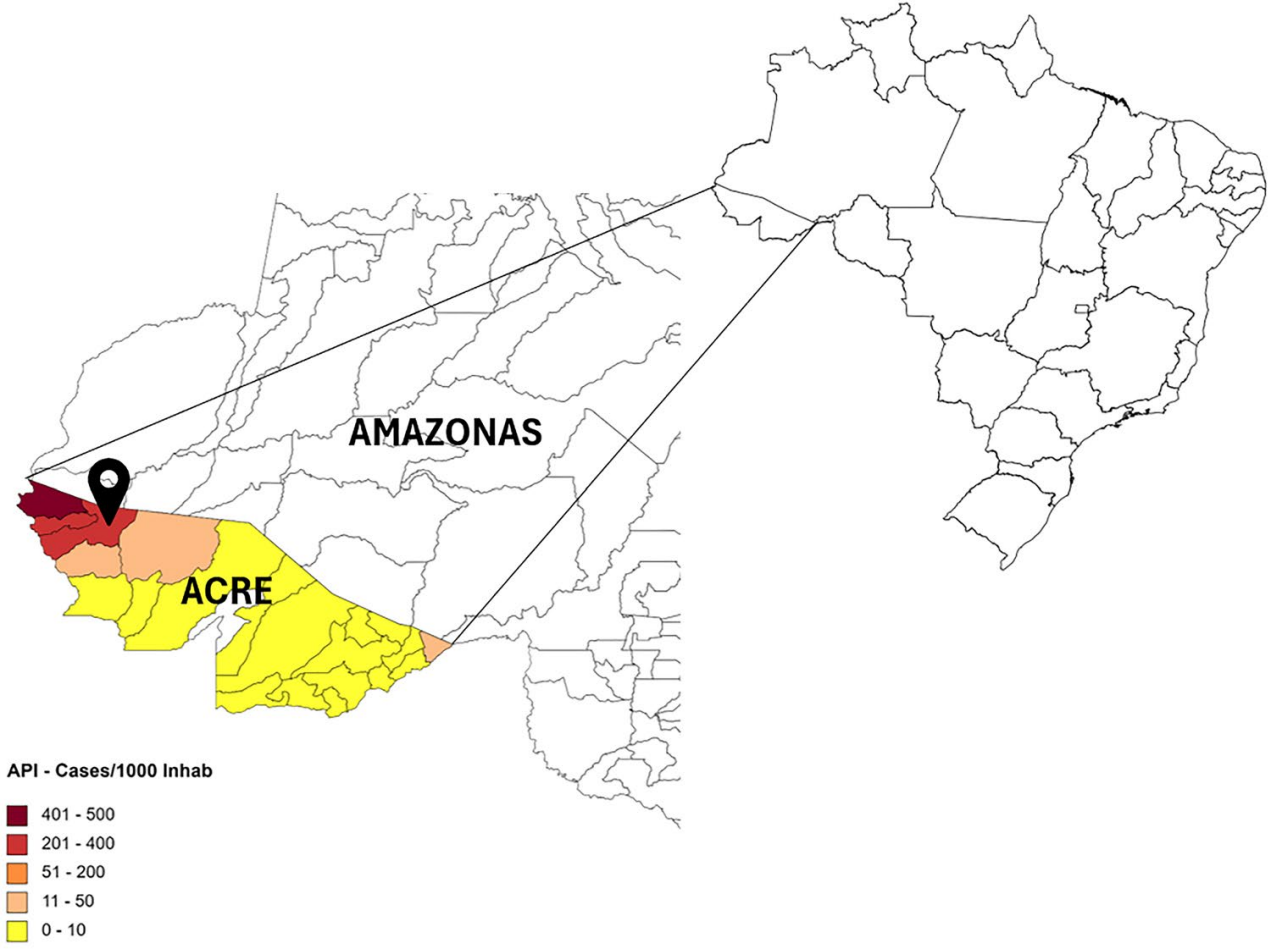
Annual parasite index (API) in Acre State in 2014. Cruzeiro do Sul municipality is indicated by the arrow (Map generated by the authors with the software https://www.mapchart.net/—Data source SIVEP—Malaria).

This study was performed in CZS (7° 37′ 51″ S, 72° 40′ 12″ W), in the northwest region of Acre State, in northern Brazil (Fig. 4). In December 2007, 3000 rectangular and conical PermaNet 2.0 (Vestergaard, Lausanne, Switzerland) deltamethrin-treated LLINs were distributed, and in December 2011, 27 rectangular and 28 conical LLINs were randomly selected by the local health authorities for the evaluation of their physical integrity and insecticidal efficacy after 4 years of use. The LLINs in the present work were the result of the first distribution of this vector control measure in Brazil. The collection of mosquito nets was carried out 4 years after distribution in an attempt to adapt to WHO standards and according to the possibility of the Brazilian Ministry of Health.

### Physical integrity analysis

This analysis was based on a previously described protocol of Killian^27^ with modifications. LLINs were hung on a metal frame to facilitate the counting of holes. Hole sizes were categorized according to the following criteria: size 1 – hole with size smaller than the tip of index finger; size 2 – hole that permits the entrance of the tip of index finger but not a fist (≥ 1 cm and < 8 cm); size 3 – hole that permits the entrance of the closed fist (≥ 8 cm) and a proportionate holes index (pHI) based on the number of holes per category (1, 2 and 3) was calculated. The location of the holes was also recorded to assess which panels of the bednet were most affected as well as which locations on each panel incurred the most physical damage^13^.

### Insecticidal efficacy

**Figure 5:**
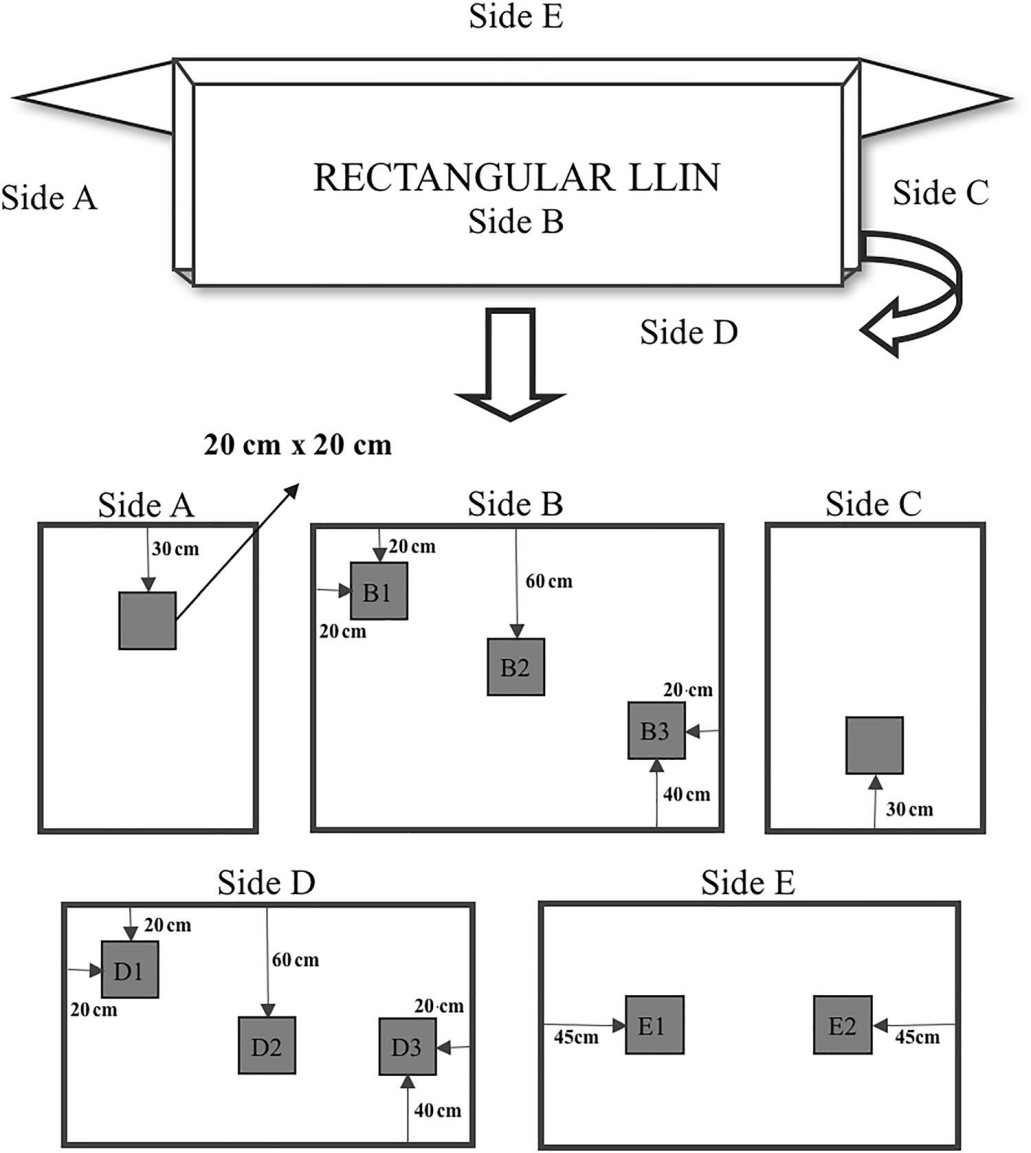
Sampling scheme of the rectangular LLINs. Squares indicate where samples were taken for bioassay and chemical analyses.

**Figure 6:**
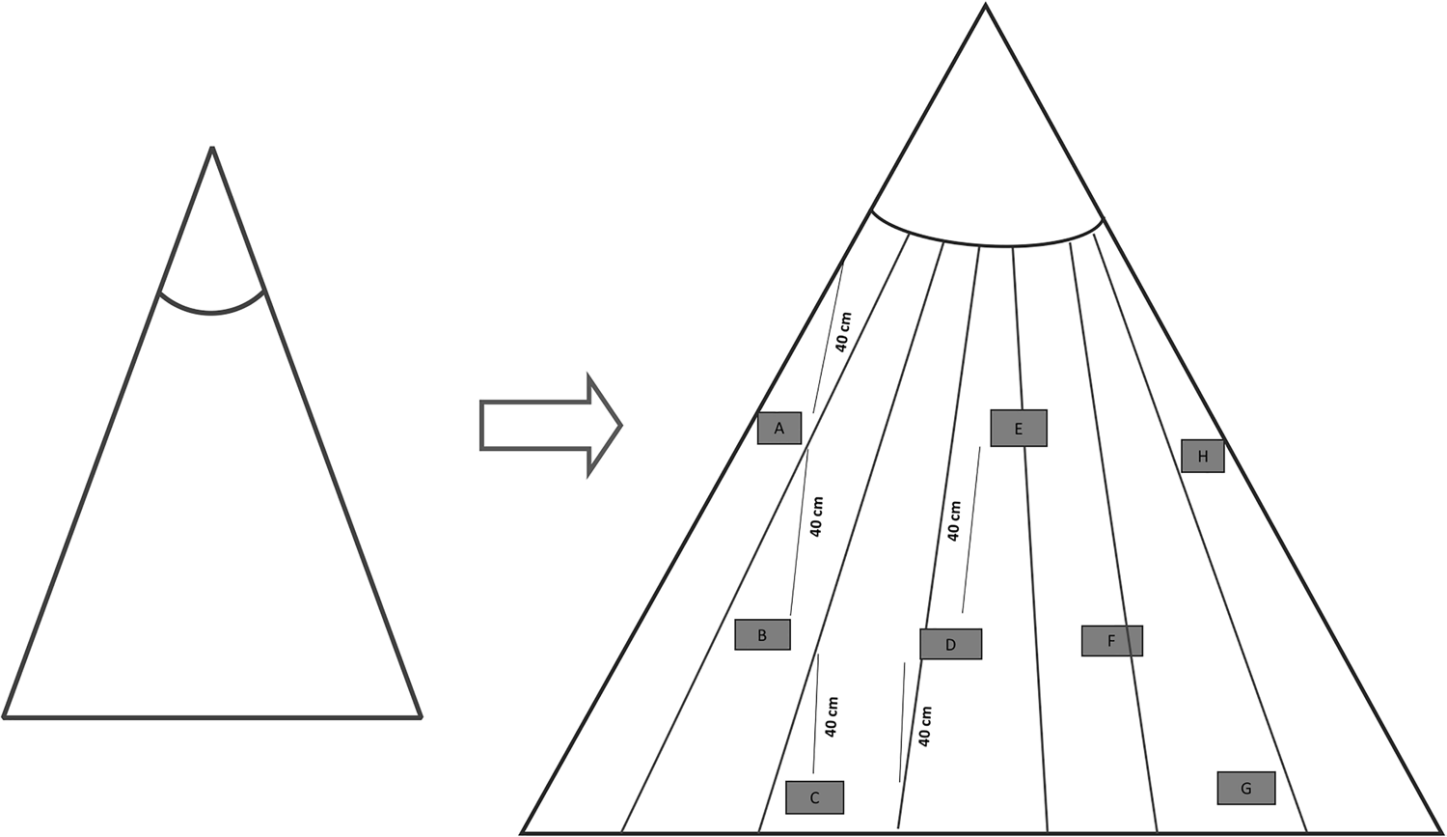
Sampling scheme of the conical LLINs. Squares indicate where samples were taken for bioassay and chemical analyses.

Based on the WHO cone bioassay test (WCT) protocol^28^, 10 pieces (20 × 20 cm) of each LLIN were evaluated, using 1 cone per piece (Figs. 5, 6). For the cone bioassays, wild-caught were used from Peixe-Boi municipality, Pará state, which are continuously monitored by our group using the CDC bottle bioassay and remain susceptible to the insecticides using the discriminating dose as recommended at Brogdon and Chan^29^. In each cone, five adult female, unfed, insecticide-susceptible An. darlingi were introduced and exposed for 3 min. At the end of this time, mosquitoes were transferred to a holding cup, where they were maintained and provided 10% sugar solution and mortality was recorded after 24 h. For each bioassay, one control was performed by exposing mosquitoes to a piece of non-impregnated bednet. The mortality index by bednet piece was determined by calculating the proportion of dead mosquitoes. For LLINs to be considered functional, they should result in mortality ≥ 80%^28^.

### Surface insecticide quantification

The cyanopyrethroid field test (CFT) was used to measure surface levels of deltamethrin on 5 pieces of LLIN out of the same 10 used for the cone bioassays^13^. From the rectangular nets, the pieces were obtained from sides A, B2, C, D2 and E2, and on conical nets from sides A, C, D, G, and H (Figs. 5, 6). In brief, for each net piece, two 13-mm diameter Whatman 597 filter paper disks were wiped using magnets (magnetizing force = 35,000 Oersted) 30 times across 90 mm of net material on both the outer and inner surfaces. The amount of deltamethrin on both filter papers was measured by comparison with filter paper disks containing known quantities of deltamethrin (calibration standard). The sample disks and the calibration disks containing known amounts of deltamethrin were placed in 24-well polystyrene flat-bottomed tissue culture plates. A 0.2 ml aliquot of a solution containing 30 mg/ml of 1,2-dinitrobenzene and 4-nitrobenzaldehyde dissolved in methyl cellosolve (2-methoxyethanol) was added to each well. After allowing the disks to soak in the reagent for five minutes, the colorimetric reaction was activated with the addition of 0.05 ml 0.4 N sodium hydroxide. The reaction was allowed to proceed for five minutes whereupon the intensity of the purple color was recorded by digital photography using a standard digital camera. Deltamethrin concentrations were determined by comparing the color intensity of the sample disks to the calibration standards using image analysis techniques as described previously^13^.

### Statistical analyses

Descriptive statistics were used to summarize the physical and chemical data from the analyzed LLINs. For the comparison between the damage found on the two LLIN types (rectangular and conical), t-tests (Mann–Whitney Rank test and Kruskal–Wallis test) and ANOVAs (nonparametric Friedman test), with Sidak's multiple comparisons tests were conducted. The chi-squared test was used to compare the observed mortality from the WCT and the amount of insecticide detected by CFT and linear regression was used to test the relationship between bioassay mortality and surface insecticide levels. The statistical tests were performed using Graphpad Prism version 7.00 for Windows (GraphPad Software, La Jolla California USA).

## Conclusion

The LLINs after 4 years of use acted primarily as a physical barrier because many were no longer sufficiently effective as a chemical barrier. The value of the colorimetric test cut-off point of 0.35 µg/sample of insecticide and insecticide resistance among *Anopheles* species should be re-evaluated in countries like Brazil with mosquito mortality proportions < 80% in the majority of its bioassays. Additional evaluations of this nature will be useful to guide LLIN distributions and accompanying community engagement activities in Brazil.

### Supplementary Information

Supplementary Table 1.

Supplementary Table 2.

## Data Availability

All data generated or analysed during this study are included in this published article and its supplementary information files.

## Abbreviations

LLINs: Long-lasting insecticidal nets
CZS: Cruzeiro do Sul
CFT: Cyanopyrethroid field test
CDC: Centers for disease control and prevention
SIVEP: Sistema de Informações de Vigilância Epidemiológica
API: Annual parasite index
WCT: WHO cone test
